# Supplementary material for: Effectiveness of a Multifaced Antibiotic Stewardship Program: A Pre-Post Study in Seven Italian ICUs
Source: J Clin Med. 2022 Jul 28;11(15):4409. doi: 10.3390/jcm11154409 (PMC9369193; doi:10.3390/jcm11154409)

Supplementary Materials

1. List of ICUs 1
2. ICU characteristics 1
3. Survival analysis for median duration of antibiotics treatments 3
4. Indicators - comparison among ICUs 4
   a. Frequency of patients with MDR infections 4
   b. Inappropriateness of antibiotics by penetration into the site of infection 5
   c. Inappropriateness of antibiotics by microorganism resistance pattern in empirical therapy 5
   d. Inappropriateness of antibiotics by microorganism resistance pattern in targeted therapy 6
   e. Use of carbapenems 6
   f. Average ICU Length of stay 7
   g. ICU Mortality 7

1. List of ICUs

- Ospedale Alessandro Manzoni di Lecco
- Presidio Ospedaliero San Leopoldo Mandić di Merate
- Ospedale San Giovanni Bosco di Torino
- Ospedale Alta Val d'Elsa di Poggibonsi
- Ospedale Maggiore di Bologna
- Ospedale Maurizio Bufalini di Cesena
- Ospedale Civile San Valentino di Montebelluna

2. ICU characteristics

ICU	1	2	3	4	5	6	7
Beds	11	5	12	6	8	17	5

ICU	1	2	3	4	5	6	7
Patients	1145	401	918	532	885	1841	568
Patients/month	52	18	38	24	37	77	24
Elective surgical (%)	8.4	29.7	18.1	13.8	26	28.9	22.4
Emergent surgical (%)	29.5	22.4	35.9	14.9	28.7	24.8	28.4
Medical (%)	62.2	47.9	45.9	71.3	45.3	46.4	49.2
Trauma (%)	50.5	12.3	12.9	6.9	19.7	32.9	16.9
Micro	external	external	external	external	internal	internal	external
Surveillance microbiological cultures - pre intervention	all patients at ICU admission (rectal swab)	no	all patients at ICU admission (recta and throat swabs, tracheal aspirate, urine culture)	subgroup of patients at ICU admission (rectal swab)	subgroup of patients at ICU admission and during stay (rectal swab)	all patients at ICU admission and subgroup of patients during stay (rectal swab)	all patients at ICU admission (recta and throat swabs)
Surveillance microbiological cultures - post intervention	all patients at ICU admission (rectal swab)	all patients at ICU admission and during stay (rectal swab, tracheal aspirate)	all patients at ICU admission (recta and throat swabs, tracheal aspirate, urine culture)	subgroup of patients at ICU admission (rectal swab	all patients at ICU admission and during stay (rectal swab)	all patients at ICU admission and subgroup of patients during stay (rectal swab)	all patients at ICU admission (recta and throat swabs)

3. Survival analysis for median duration of antibiotics treatments

The indicators on the duration of antibiotics treatments are constructed to measure when the antibiotic treatment (either prophylaxis or empirical therapy) are suspended. The focus is not on the real duration of the treatment, but on the decision made by the prescribers.

According to this aim, we construct a Kaplan-Meier curve where the outcome is the decision of interruption of the antimicrobial treatment made by an intensivist. We censor all those treatments for which the decision is not made during the ICU stay, because the patient either is discharged or dies with an ongoing treatment.

The median duration t_{median} is derived from the curve, as the time when the curve crosses 50%, e.g., the time before which 50% of treatments are interrupted due to a clinical decision, as illustrated in the following Figure. Analogously, the confidence interval (t_{low}, t_{up}) is derived as the time points where the horizontal line at 50% intersects the boundaries of the confidence band of the survival curve.

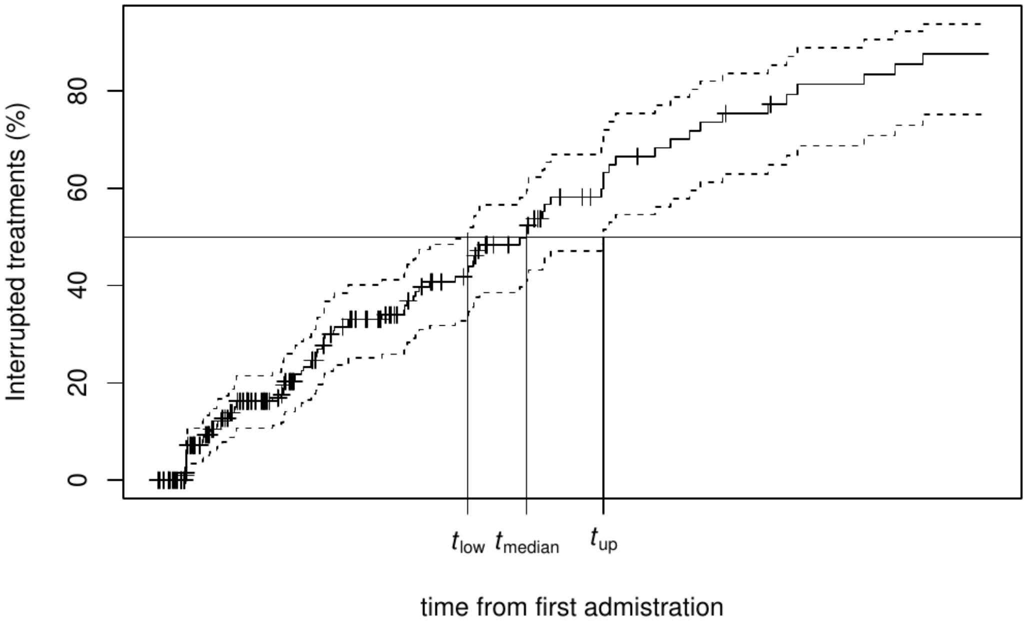

4. Indicators - comparison among ICUs

a. Frequency of patients with MDR infections

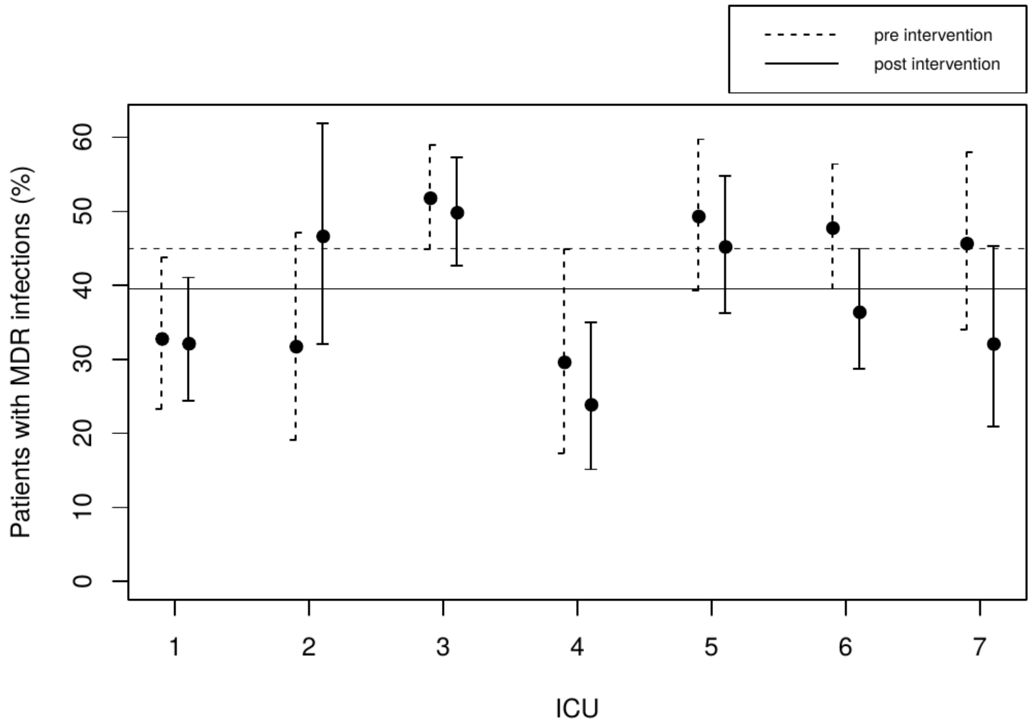

b. Inappropriateness of antibiotics by penetration into the site of infection

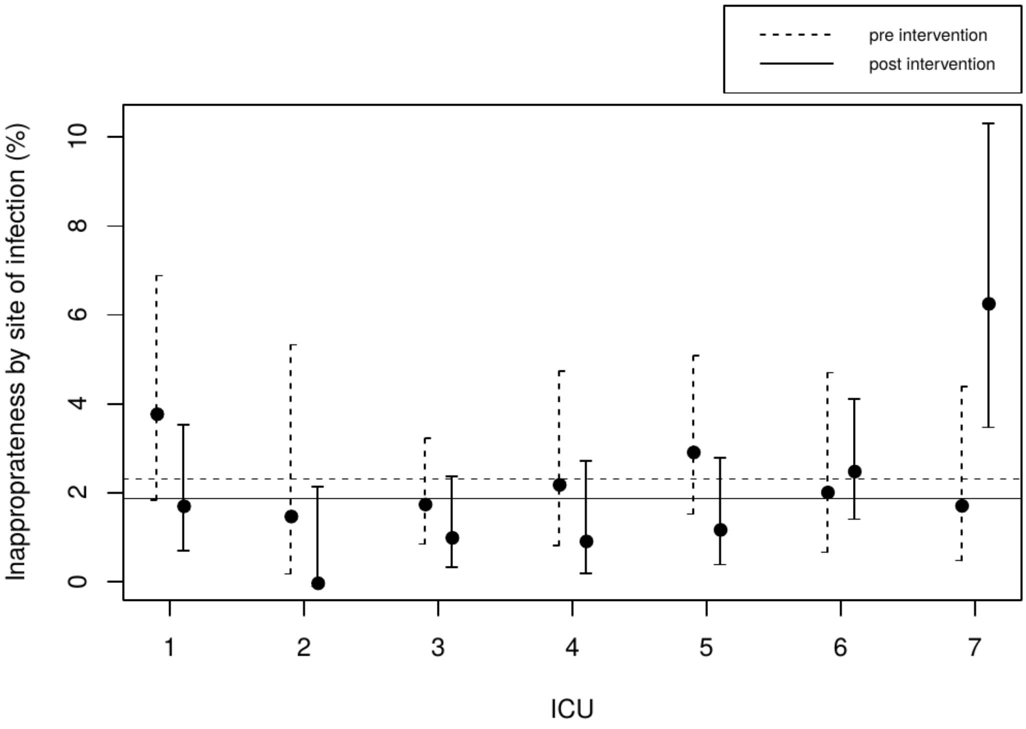

c. Inappropriateness of antibiotics by microorganism resistance pattern in empirical therapy

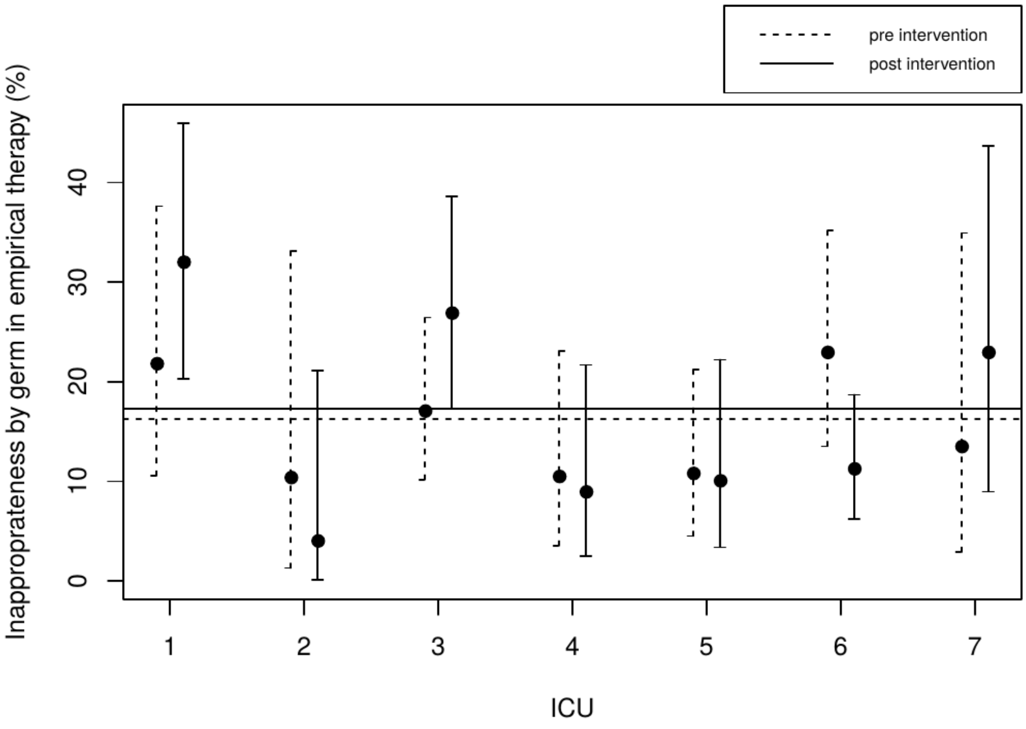

d. Inappropriateness of antibiotics by microorganism resistance pattern in targeted therapy

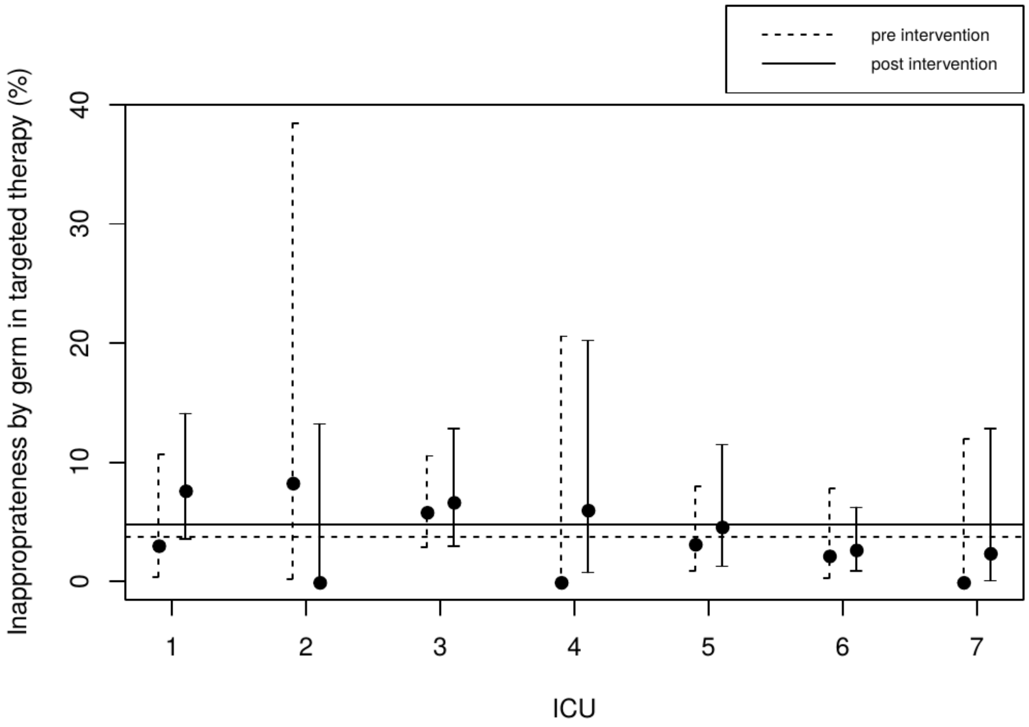

e. Use of carbapenems

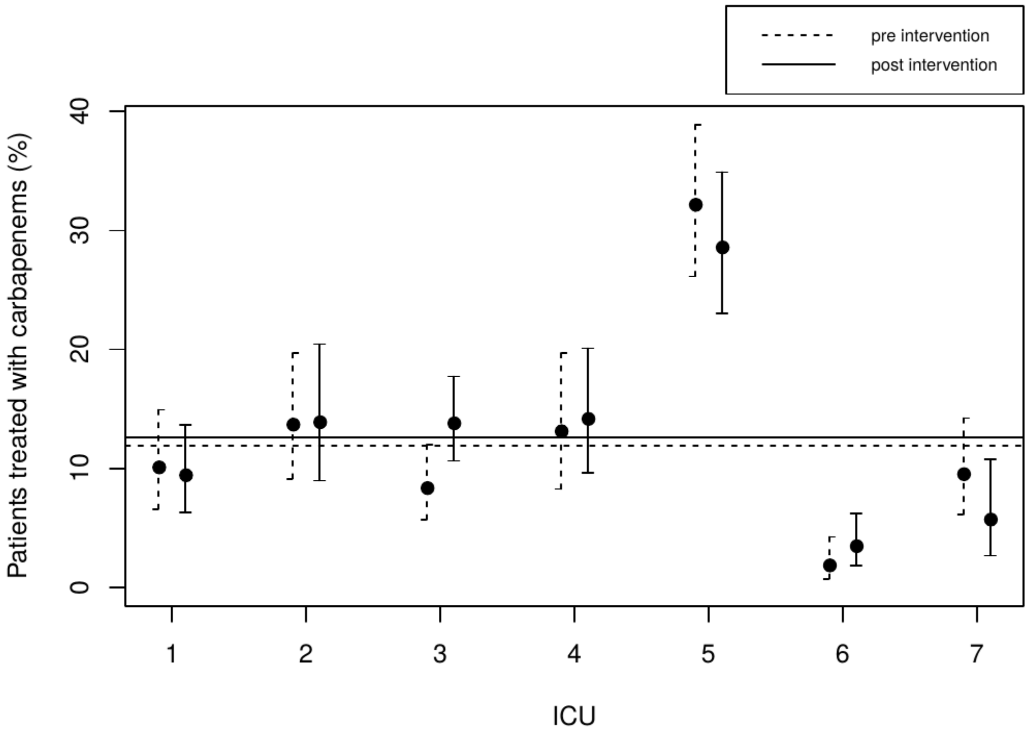

f. Average ICU Length of stay

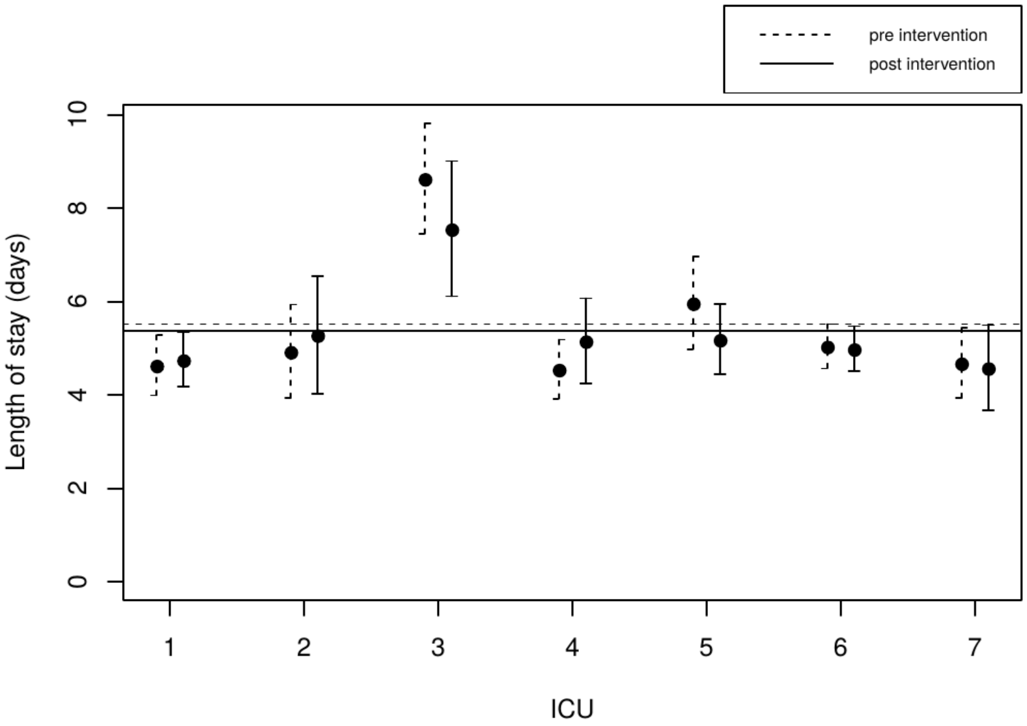

g. ICU Mortality

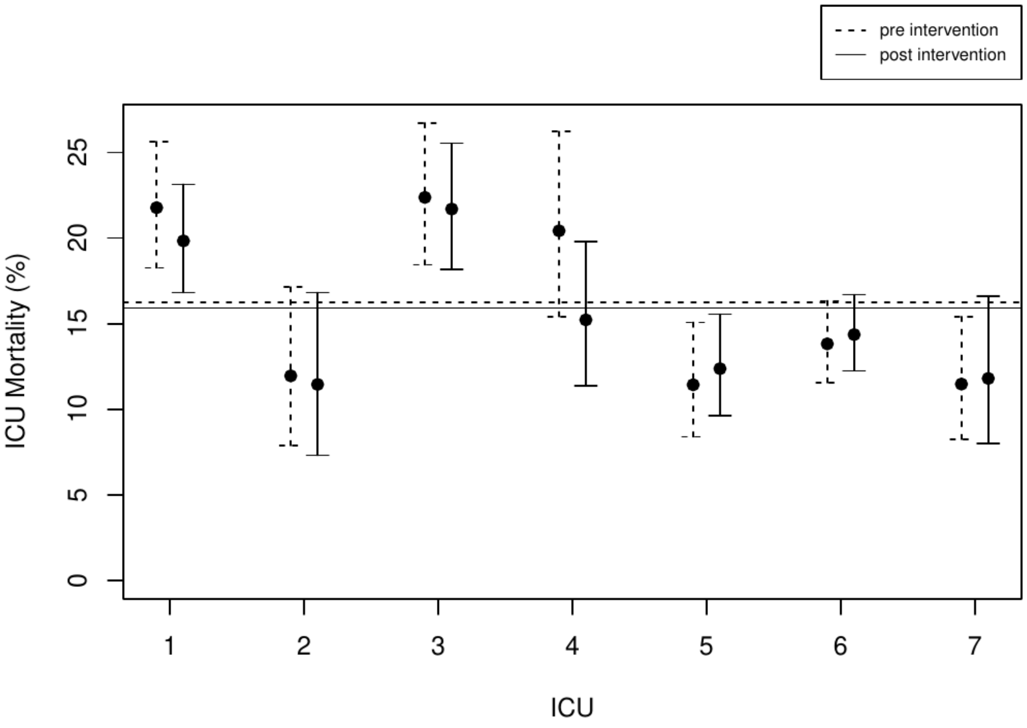

Supplement: Supplementary file 1 [file jcm-11-04409-s001.zip › jcm-1756467-supplementary.pdf]
